# The Influence of Individual, Social and Physical Environment Factors on Physical Activity in the Adult Population in Andalusia, Spain

**DOI:** 10.3390/ijerph7010060

**Published:** 2010-01-05

**Authors:** Julia Bolívar, Antonio Daponte, Miguel Rodríguez, José Juan Sánchez

**Affiliations:** Andalusian School of Public Health (Escuela Andaluza de Salud Pública), Cuesta del Observatorio 4, 18080 Granada, Spain; E-Mails: antonio.daponte.easp@juntadeandalucia.es (A.D.); miguel.rodriguez.easp@juntadeandalucia.es (M.R.); josejuan.sanchez.easp@juntadeandalucia.es (J.S.)

**Keywords:** physical activity, social environment, socio-economic factors, environment, Spain

## Abstract

A person’s physical and social environment is considered as an influencing factor in terms of rates of engagement in physical activity. This study analyses the influence of socio-demographic, physical and social environmental factors on physical activity reported in the adult population in Andalusia. This is a cross-sectional study using data collected in the Andalusia Health Survey in 1999 and 2003. In addition to the influence of the individual’s characteristics, if there are no green spaces in the neighbourhood it is less likely that men and women will take exercise (OR = 1.26; 95% CI = 1.13, 1.41). Likewise, a higher local illiteracy rate also has a negative influence on exercise habits in men (OR = 1.39; 95% CI = 1.21, 1.59) and in women (OR = 1.22; 95% CI = 1.07, 1.40). Physical activity is influenced by individuals’ characteristics as well as by their social and physical environment, the most disadvantaged groups are less likely to engage in physical activity.

## 1. Introduction

There is a large amount of evidence that shows that physical activity benefits a person’s health. Physical activity can be helpful for health promotion, rehabilitation and the prevention of different diseases such as heart disease, high blood pressure, diabetes mellitus and osteoporosis, and can of course be extremely important for obese patients [1,2]. Sedentariness is one of the most important cardiovascular risk factors, and cardiovascular diseases were the leading cause of death in Spain in 2007, representing 32.2% of all deaths [3].

Over recent years, an increased number of studies have been carried out which examine the factors which influence physical activity, with particular emphasis on the social and physical environment [4,5]. Such studies not only take into consideration the characteristics of individuals, but also those of the environment in which they live.

The influence of social inequalities on physical activity has now been widely researched, and work has been carried out to examine the effects of the social status and environmental context of the studied individuals on their exercise habits [6–8]. Thus, the influence of the social class of individuals [9] and the socio-economic characteristics of the geographic area in which they live [10] is now examined in order to demonstrate that the principal factors influencing health-related habits such as physical activity are environmental in nature and combine to create an unequal socio-economic background for those involved [11–13]. These factors lead to disparities in the level of physical activity carried out and consequently to disparities in levels of health.

Even more recently studies have been carried out into the influence of the physical environment. In the literature the concept of physical environment has been defined as the existence of and physical accessibility to centres such as gyms, swimming pools and leisure centres; “informal spaces” that form part of a neighbourhood’s facilities such as open public spaces, and the layout and use of buildings; or aspects regarding traffic, safety and attractiveness of neighbourhoods and local areas [9,14–16]. These studies have not simply used objective measurements such as the existence, accessibility or proximity of such facilities. They have also shown that the perceptions and opinions of individuals about their environment are related to the extent to which they engage in physical activity [6,15,17,18]. Furthermore, it has even been shown that people who believe that their environment is suitable for taking physical exercise are more likely to perceive that they have a good level of health [2]. Studies conducted throughout Europe have found that Spain is one of the countries where the least physical activity is carried out, where people’s attitudes towards exercise are more negative and where individuals feel that their environment offers them few opportunities [19,20]. Despite this, no studies have been undertaken in Spain regarding the relationship between the physical and social environment and rates of physical activity.

There are major regional socio-economic disparities in Spain. Andalusia is a large region in the south that accounts for just over 18% of the country’s total population, and it is one of the most disadvantaged regions in terms of unemployment and income, amongst other factors. These disparities affect the health-related habits such as physical activity and the health status of the region’s population [21]. According to data from the National Health Survey [22], the percentage of the region’s inhabitants who engage in physical activity in their free time is one of the lowest of all of Spain’s regions.

For this reason, and in view of the low number of studies published here which take into account the characteristics of individuals as well as their physical and social environment, the aim of this work was to analyze the influence of the characteristics of individuals and their physical and social environment on their rates of engagement in physical activity. The study used data collected from inhabitants of Andalusia aged 16 and over. The objective of this study was to analyze the influence of the characteristics of individuals and of their physical and social environment on rates of physical activity according to data collected from the adult population in Andalusia.

## 2. Methods

Cross-sectional study using data regarding the adult population (aged 16 and over) living in Andalusia, excluding people living in care, collected by the Andalusia Health Survey in 1999 and 2003 (N: 6425 men and 6768 women). The Andalusian Government designed and directed both surveys and the sampling methodology used was similar in both cases. Its sampling design was probabilistic, stratified and multi-stage [23]. Adding both surveys we had repeated measurements in different samples.

### 2.1. Dependent Variable: Physical Activity during Free Time

Self-reported physical activity during free time. Interviewees were asked about the type of physical activity that they carried out in their free time. Possible answers were: 1 (I don’t do any exercise. I spend almost all of my free time in a sedentary manner by reading, watching TV, going to the cinema, *etc.*); 2 (Some physical activity or occasional sports, such as walking, bike rides, gardening, low-intensity gym, *etc.*); 3 (Regular physical activity several times a month, such as tennis, gym, running, swimming, cycling, *etc.*); 4 (Physical training several times a week, including physical activity several times a week). Values were split into two groups—those who said their free time was spent in a sedentary manner and those who carried out physical activity (engaging in some activity occasionally, regularly or as training).

### 2.2. Independent Variables

The socio-demographic variables considered were: sex, age, marital status, whether or not participants had children aged 15 and under; educational level, social class: Class I (Directors or university lecturers), Class II (Civil servants, personal services, self-employed people, supervisors), Class III (Skilled and semi-skilled labourers), Class IV (Unskilled labourers) [24]; employment status; self-rated health status; Obesity, following Quetelet’s body mass index: no (<30), yes (≥30); and smoking. All of these variables are characteristics of the individuals.

With regard to the physical environment, we considered the individual’s own perception of his/her neighbourhood environment. The following data were collected from the Population and Housing Census for 2001, conducted by the National Institute of Statistics [25].

There are sufficient green spaces in your neighbourhood; noise from outdoors annoys you; the air is highly polluted in your neighbourhood; bad smells come into your house from the outside; your neighbourhood is affected by an industry; the quality of your neighbourhood environment is: good (very good or good), bad (poor, bad or very bad).

Finally, the group of variables related to the social environment refers to the socio-economic characteristics of the municipality. The economic level of the municipality was gauged using the family income index available per inhabitant, estimated according to geographical area, and it was obtained from the Spanish Annual Economic Report [26]: Low (Up to 8,300 euros), Middle (8,300–10,200 euros), and High (10,200–12,100 euros).

The size of the municipality and its illiteracy and unemployment rates were obtained from the Population and Housing Census for 2001, conducted by the National Institute of Statistics [25]. Tertiles were calculated for illiteracy and unemployment by dividing cases into three equal-sized groups. Variables were thus categorised according to whether the municipality had a low (first tertile), medium (second tertile) or high (third tertile) rate of illiteracy or unemployment.

### 2.3. Statistical Analysis

A bivariate analysis was performed to compare distribution of activity between men and women, using the chi-square test. An age-adjusted prevalence of physical activity during free time was analysed with a direct method of standardization, using the spanish standard population. A bivariate analysis of physical activity was also performed in comparison with the other variables, estimating risk of sedentariness through odds ratio (OR), using a 95% confidence interval (CI 95%). Multivariate regression models, using binary logistic regression, were analysed for men and women using the data collected regarding engagement in physical activity during free time as the variable (0: Physical Activity, 1: Sedentary). The statistical package SPSS 11.5 for Windows was used to carry out the analyses.

## 3. Results

Women engage in less physical activity during free time than men, at rates of 41.9% and 51.1% respectively (p < 0.001) in all age groups. The highest frequency is observed in the 16–24 year-old group in both sexes (Table 1). Men who have not attended school engage in the least physical activity during free time, 36.7%, compared with 64.9% of men with graduate studies (p < 0.001). This is the same case for women: 26.8% *vs.* 58.0% (p < 0.001). With regard to employment status, students (77.2%), followed by the unemployed (52.2%) are those who engage in the most physical activity (p < 0.001). The same pattern is found in women, with 57.7% of female students and 49.1% of unemployed women engaging in physical activity (p < 0.001).

With reference to health-related lifestyles, men and women who perceive their level of health to be good (54.4% of men and 47.1% of women) and are not obese (54.5% of men and 45.7% of women) tend to carry out more physical activity. With regard to smoking, male non-smokers carry out the most physical activity (61.2%), followed by ex-smokers, 49.1% (p < 0.001). In women, ex-smokers carry out the most physical activity (50.4%), followed by smokers (44.2%).

With regard to physical environment, men who believe that there are sufficient green spaces in their neighbourhood carry out the most physical activity (54.9%), (p < 0.001). The same is true for women (44.2%) (p < 0.001). There are no statistically significant differences for the remaining variables related to physical environment in either sex.

The social environment also appears to be an influencing factor with regard to physical activity, since men who live in areas with over 100,000 inhabitants carry out the most physical activity (53.0%) (p < 0.05). This is also true of men who live in municipalities with a high economic level, and those who live in municipalities with a low illiteracy rate. For women, living in municipalities with a low unemployment rate and low illiteracy rate appears to be statistically significant in relation to engaging in physical activity.

**Figure 1.**
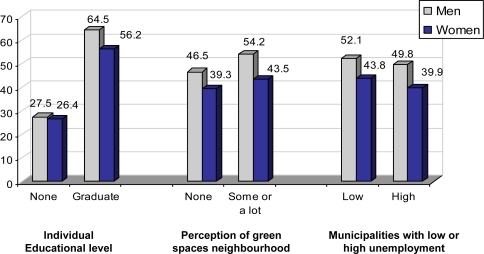
Age-adjusted Prevalence of Physical Activity in relation to individual, physical and social environment.

Disparities are observed after age adjustment, and those who have not attended school, those who live in areas without green spaces and those who live in municipalities with high unemployment rates are at the greatest disadvantage. This is especially so in the case of women (Figure 1).

Table 2 shows the bivariate analysis of sedentariness with each variable adjusted by age. People who belong to Class IV have a higher probability of being sedentary in comparison with those who belong to Class I. This higher probability stands at 61% in men and 63% in women (OR = 1.61; 95% CI = 1.31, 1.99 and OR = 1.63; 95% CI = 1.33, 2.00). This probability is higher amongst those who have not attended school when compared with those who have graduate studies (OR = 2.54; 95% CI = 2.06, 3.13 and OR = 2.99; 95% CI = 2.41, 3.70 respectively), and therefore the most disadvantaged groups are those who carry out the least physical activity.

Employment status is statistically linked to sedentariness and as a result homemakers are 43% more likely to be sedentary than female students (OR = 1.43; 95% CI = 1.17, 1.75). Working men are almost three times more likely to be sedentary than students (OR = 2.82; 95% CI = 2.31, 3.46).

Less than good health perceived and being obese have a negative impact on rates of physical activity in men and women. This also applies to smokers. It should be noted that in the case of women, ex-smokers have a 22% lower probability of being sedentary than non-smokers (OR = 0.78; 95% CI = 0.64, 0.96).

In the physical environment dataset, men and women’s perception of the lack of green spaces in their neighbourhood is significant (OR = 1.36; 95% CI = 1.23, 1.50 and OR = 1.21; 95% CI = 1.10, 1.34 respectively) in terms of predicting sedentariness.

The social environment is linked to sedentariness, and this is particularly true in the case of men. Living in municipalities of over 100,000 inhabitants has a protective effect in comparison with living in small municipalities (OR = 0.87; 95% CI = 0.76, 0.99). Similar results are observed in men who live in municipalities with a high economic level, who are 21% more likely not to be sedentary than those who live in municipalities with a low economic level (OR = 0.79; 95% CI = 0.68, 0.91). Municipalities with higher rates of illiteracy and unemployment have an increased probability of sedentariness, especially in the case of illiteracy.

Table 3 shows three multivariate models based on the three major groups of characteristics: individual, and physical and social environment. In the first group of variables (model 1), educational level and social class have been used as indicators of social status. Employment status, the presence of children under the age of 15 and all health variables continue to be significant influencing factors on rates of sedentariness in men. In women, in addition to marital status, having children is not linked to sedentariness. Thus, for both sexes, poor health and smoking are both linked to a higher probability of sedentariness, as well as working and not having academic qualifications. The latter variable is particularly important in the case of women (OR = 2.71; 95% CI = 2.07, 3.55).

In the physical environment dataset (model 2), the presence of green spaces is of note. Men who perceive that there are no green spaces in their neighbourhood are 37% more likely to be sedentary than those who perceive the opposite. In the case of women, this probability stands at 19% (OR = 1.37; 95% CI = 1.24, 1.52 and OR = 1.19; 95% CI = 1.08, 1.32 respectively).

With regard to the social environment (model 3), the illiteracy and unemployment rates of a municipality have a negative effect on the rates of physical activity for both sexes, and higher rates of sedentariness are observed in municipalities with medium and high levels of illiteracy and unemployment. This is the same case for municipalities with a low economic level, although it is not statistically significant.

After analysing the different groups of variables separately, and in order to further the analysis, a final multi-variate model was drawn up (Table 4), which incorporates the characteristics of the individuals as well as those of their physical and social environment. All of these variables were significant in the earlier analyses because of their links with sedentariness. By entering these variables into this final model it is apparent that the same trends and similar values continue to exist. This serves to prove the importance of using frameworks which take into account a number of factors which can influence the behaviour of individuals. The most important factors which influence sedentariness are the educational level and social status of individuals (especially in the case of women), the presence of green spaces, and the socio-economic level of the municipality in which they live. The results show evidence of disparities and demonstrate the major influence of the living environment on the behaviour of individuals.

## 4. Discussion

Research into rates of physical activity has traditionally been focused on individual factors, and this is partly because of the difficulties involved in examining social and structural influences. Over recent years, studies have been carried out which examine the influence of environmental factors on the engagement of individuals in physical activity [27]. This study provides data regarding Andalusia, a large region in the south of Spain, and identifies the individual, social and physical environmental factors which have an influence on physical activity. The importance of this study lies in its emphasis on the role of the social and physical environment as a key factor influencing rates of physical activity, given that Andalusia is one of the regions with the lowest socio-economic levels in Spain and where the least physical activity is carried out [28,29].

We found that there were higher rates of sedentariness amongst women, smokers, obese people, and those who perceive their health as poor, and that age also has a negative effect on rates of physical activity. These results are consistent with those of other studies [10,30,31]. The most disadvantaged social classes and people with the lowest educational level are more sedentary. On the one hand, reduced economic resources may impose a limit on paying gym membership fees or playing sports during free time, and on the other hand, high levels of physical activity during the working day (in the case of labourers) may prevent these people from taking exercise in their free time [13]. Another explanation for the reduced level of exercise in these groups is that it is less likely that they understand and heed messages regarding the negative effects of sedentariness, as shown in studies on other health-related behaviours, such as smoking [32].

Furthermore, both men and women’s perception of green spaces in their neighbourhood has an influence on rates of physical activity. With regard to physical activity, social class is still prevalent in the physical context, since people from higher social classes and with higher qualifications who consider that their neighbourhoods have many green spaces engage in more physical activities (data not shown).

Some studies reveal that the most disadvantaged groups have a more marked perception that their neighbourhoods are not attractive, have more traffic and are more stressful for physical activity [9]. Physical environmental factors such as perception of safety in a neighbourhood, its attractiveness, the presence of passable pavements, open public spaces, leisure centres and green spaces, have been found to have a major influence on rates of physical activity in other studies, after adjustment according to socio-demographical variables such as age, educational level, ethnic groups, *etc.* [15,16,33]. These studies suggest that action taken based on environmental innovations could favor more active lifestyles [17,19] and that in order to increase physical activity, it is necessary to consider the way in which space is used [5,34].

Services and activities organised in “informal spaces” (open spaces, green zones, *etc.*) should be considered important components of a neighbourhood’s facilities that can help to promote physical activity. The reason for considering this paradigm is that neighbourhood and environmental actions can target a higher number of people at a potentially lower cost per person than actions which target individuals or groups, thus reaping a greater benefit for public health [6,33]. Furthermore, the available space could be multi-functional e.g., in the different life-stage of the population, the local needs, *etc.* Such actions are therefore necessary and we should study how to strengthen their appeal, security, accessibility and nearness. However, they are not sufficient in themselves to increase the recommended levels of physical activity in a neighbourhood. Healthy environments are directly related to the development of public policies and these policies should not be exclusive to the health sector [2,35].

In our study we incorporated the socio-economic, unemployment and illiteracy indicators of municipalities, and found that the most deprived municipalities have the highest levels of sedentariness. Individuals who live in depressed areas are more likely to be physically inactive than those who live in more advantaged areas [27]. This is partly due to a reduced social expenditure in programmes and services, and also to a series of processes that arise and trigger a vicious circle in the neighbourhood. This theory explains the positive link between the socio-economic level of a person’s environment and his or her level of physical activity [36]. A recent study in Spain showed that, after adjustments had been made for socio-economic and other individual characteristics, the effect of the economic situation of a province no longer influences the rates of physical activity in men, although this is not so in the case of women [10].

The use of specific aspects of activity, such as activity type, where it takes place and how it is measured, may affect the distribution of disparities, as well as the environmental characteristics that play a part in the link between the socio-economic level of the area and rates of physical activity [34].

In this study we have used statistics based on the perception of participants with regard to physical activity and the existence of green spaces in the neighbourhood. The validity of such methods has been proved by other studies that have also used this type of measurement [17]. Furthermore, concepts such as “neighbourhood” and “green space” can be ambiguous, as they are constructs [37] which may be interpreted differently according to the social and cultural beliefs of individuals. As a result, qualitative studies could help us to gain a better understanding of factors that influence rates of physical activity [7].

Regarding the analysis, we have explored multilevel models too, for the multivariate final model (data not shown). The values of rho coefficient, which measures the percentage of total variability explained by the second level, were low for all the models. The percentage of variability explained by the aggregation level (municipalities) was less than 6% of the total variability in all cases. In addition, the changes in the OR were not important, and the significances found did not change, so our conclusions do not vary when considering the multilevel structure.

## 5. Conclusions

In conclusion, rates of physical activity in Spain are influenced by our social and physical environment. The influence of social class means that members of disadvantaged groups are less likely to engage in physical activity. Furthermore, women are less active than men. The presence of green spaces in neighbourhoods is an influencing factor. We need to improve our knowledge of the mechanisms which affect the most disadvantaged groups, incorporating a gender perspective in view of the lower rates of physical activity amongst women, and a physical and social environment perspective, so that actions and interventions to promote physical activity and healthy lifestyles that are carried out in any area do not result in the same disparities as before or create further disparities.

## Figures and Tables

**Table 1.** Distribution of variables by sex. Physical activity during free time.

	**Men**					**Women**					
	**Physical Activity**		**Sedentary**			**Physical Activity**		**Sedentary**			
	**N**	**%**	**N**	**%**	P PA*	**N**	**%**		**%**	P PA*	**P sex**
**N**	**3261**	(**51.1)**	**3122**	(**48.9)**	*	**2813**	(**41.9)**	**3895**	(**58.1)**	*	*
**Age**											
16–24	851	(69.5)	373	(30.5)	*	601	(51.8)	559	(48.2)	*	*
25–44	1248	(49.6)	1268	(50.4)		1130	(45.4)	1357	(54.6)		*
45–64	706	(42.6)	950	(57.4)		703	(40.9)	1014	(59.1)		NS
≥56	456	(46.2)	532	(53.8)		378	(28.1)	965	(71.9)		*
**Educational level**											
No studies	354	(36.7)	611	(63.3)	*	416	(26.8)	1137	(73.2)		*
Primary	1338	(45.3)	1617	(54.7)		1313	(41.9)	1822	(58.1)	*	*
Secondary	980	(63.5)	564	(36.5)		640	(51.7)	599	(48.3)		*
Graduate studies	552	(64.9)	298	(35.1)		412	(58.0)	298	(42.0)		*
**Occupational Class**											
Class I (highest)	641	(59.9)	429	(40.1)	*	492	(51.4)	466	(48.6)		*
Class II	700	(54.9)	576	(45.1)		575	(48.4)	612	(51.6)	*	*
Class III	1600	(47.5)	1766	(52.5)		1329	(39.0)	2080	(61.0)		*
Class IV(lowest)	254	(46.7)	290	(53.3)		240	(37.0)	408	(63.0)		*
**Marital status**											
Single	1373	(62.8)	813	(37.2)	*	870	(50.0)	871	(50.0)	*	*
Married / couple	1746	(45.2)	2115	(54.8)		1617	(40.8)	2343	(59.2)		*
Separated, divorced, widowed	142	(42.4)	193	(57.6)		325	(32.4)	679	(67.6)		*
**Employment status**											
Employed	1565	(47.8)	1707	(52.2)	*	610	(45.0)	747	(55.0)	*	NS
Unemployed	374	(52.2)	343	(47.8)		288	(49.1)	299	(50.9)		NS
Retired, disabled	734	(45.2)	890	(54.8)		171	(31.4)	373	(68.6)		*
Homemaker	-	-	-	-		1331	(38.1)	2162	(61.9)		NS
Student	572	(77.2)	169	(22.8)		399	(57.7)	292	(42.3)		*
**Children ≤15 years**											
No	2599	(52.9)	2312	(47.1)	*	2070	(41.3)	2946	(58.7)	*	NS
Yes	662	(45.0)	810	(55.0)		743	(43.9)	949	(56.1)		*
**Self-rated Health**											
Good	2748	(54.4)	2303	(45.6)	*	2164	(47.1)	2428	(52.9)	*	*
Less than good	511	(38.5)	816	(61.5)		648	(30.7)	1463	(69.3)		*
**Smoking status**											
Smoker	1183	(43.4)	1540	(56.6)		745	(44.2)	939	(55.8)		NS
Ex-Smoker	654	(49.1)	678	(50.9)	*	212	(50.4)	209	(49.6)	*	NS
Non-smoker	1425	(61.2)	902	(38.8)		1855	(40.3)	2744	(59.7)		*
**Obesity**											
No (<30)	2742	(54.5)	2286	(45.5)	*	2174	(45.7)	2586	(54.3)	*	*
Yes (≥30)	332	(38.8)	523	(61.2)		298	(33.5)	592	(66.5)		*
**In your neighbourhood there are sufficient green spaces**											
None	1505	(47.4)	1673	(52.6)	*	1367	(39.9)	2058	(60.1)	*	*
A lot or some	1740	(54.9)	1429	(45.1)		1437	(44.2)	1813	(55.8)		*
**Noise from outdoors annoys you**											
None	1942	(51.1)	1859	(48.9)	NS	1628	(42.2)	2229	(57.8)	NS	*
A lot or some	1311	(51.2)	1251	(48.8)		1182	(41.7)	1653	(58.3)		*
**Bad smells come into your house from outside**											
None	2599	(51.2)	2479	(48.8)	NS	2147	(42.1)	2948	(57.9)	NS	*
A lot or some	652	(51.0)	627	(49.0)		662	(41.6)	931	(58.4)		NS
**The air is highly polluted in your neighbourhood**											
None	2681	(50.6)	2613	(49.4)	NS	2248	(41.6)	3154	(58.4)	NS	*
A lot or some	560	(53.9)	479	(46.1)		546	(44.0)	694	(56.0)		**
**Your neighbourhood is affected by an industry**											
None	2952	(50.8)	3038	(46.2)	NS	2550	(42.0)	3519	(58.0)	NS	*
A lot or some	291	(54.7)	241	(45.3)		247	(41.9)	343	(58.1)		*
**Quality of your neighbourhood environment**											
Good	2473	(50.7)	2401	(49.3)	NS	2115	(42.2)	2902	(57.8)	NS	*
Bad	787	(52.3)	717	(47.7)		695	(41.4)	982	(58.6)		*
**Size of municipality**											
<10,000 inhabitants	727	(48.8)	762	(51.2)		674	(42.4)	917	(57.6)		*
10,000–100,000 inhabitants	1247	(50.5)	1220	(49.5)	**	1078	(42.0)	1488	(58.0)	NS	*
>100,000 inhabitants	1287	(53.0)	1141	(47.0)		1061	(41.6)	1490	(58.4)		*
**Economic level of municipality**											
Low (< 8,300 €)	613	(46.7)	700	(53.3)		573	(40.2)	852	(59.8)		*
Medium(8,300–10,200 €)	1527	(51.5)	1438	(48.5)	*	1282	(41.4)	1814	(58.6)	NS	*
High (10,200–12,100 €)	1045	(53.1)	923	(46.9)		882	(43.1)	1164	(56.9)		*
**Unemployment in municipality**											
Low	1147	(52.7)	1029	(47.3)	NS	101	(44.4)	1253	(55.6)	**	*
Medium	1048	(49.9)	1052	(50.1)		900	(40.8)	1304	(59.2)		*
High	1066	(50.6)	1041	(49.4)		912	(40.5)	1338	(59.5)		*
**Illiteracy in municipality**											
Low	1168	(55.3)	945	(44.7)	*	1003	(45.0)	1227	(55.0)	*	*
Medium	1060	(49.6)	1076	(50.4)		879	(39.4)	1350	(60.6)		*
High	990	(48.4)	1057	(51.6)		890	(41.2)	1272	(58.8)		*

NS: Not significant: >0.05;

**: P < 0.05;

*: P < 0.001.

**Table 2.** Bivarate analysis. Odds ratio for sedentariness and variables related to characteristics of individuals and their physical and social environment, one by one, age-adjusted.

	**Men**			**Women**		
	**OR**	**95% CI**	**P**	**OR**	**95% CI**	**P**
**Educational level**						
Graduate	**1**			**1**		
Secondary	**1.15**	(0.96–1.37)		**1.33**	(1.10–1.60)	
Primary	**2.17**	(1.85–2.54)	*	**1.80**	(1.52–2.13)	*
No studies	**2.54**	(2.06–3.13)		**2.99**	(2.41–3.70)	
**Occupational Class**						
Class I (highest)	**1**			**1**		
Class II	**1.17**	(0.99–1.38)		**1.08**	(0.91–1.28)	
Class III	**1.56**	(1.35–1.80)	*	**1.55**	(1.34–1.79)	*
Class IV (lowest)	**1.61**	(1.31–1.99)		**1.63**	(1.33–2.00)	
**Employment status**						
Student	**1**			**1**		
Worker	**2.82**	(2.31–3.46)	*	**1.36**	(1.12–1.65)	**
Unemployed	**2.47**	(1.95–3.13)		**1.22**	(0.97–1.52)	
Retired, disabled	**2.07**	(1.55–2.75)		**1.53**	(1.14–2.04)	
Homemaker	-			**1.43**	(1.17–1.75)	
**Marital status**						
Single	**1**			**1**		
Married / couple	**1.67**	(1.46–1.90)	*	**1.07**	(0.94–1.22)	NS
Separated, divorced, widowed	**1.67**	(1.29–2.17)		**1.11**	(0.90–1.37)	
*Children ≤15 years*						
No	**1**			**1**		
Yes	**1.30**	(1.13–1.48)	*	**1.12**	(0.98–1.28)	NS
**Obesity**						
No (<30)	**1**			**1**		
Yes (≥30)	**1.65**	(1.42–1.92)	*	**1.34**	(1.15–1.57)	*
**Smoking status**						
Non-smoker	**1**			**1**		
Smoker	**2.02**	(1.80–2.27)	*	**1.15**	(1.01–1.29)	*
Ex-Smoker	**1.16**	(1.00–1.34)		**0.78**	(0.64–0.96)	
**Self-rated Health**						
Good	**1**			**1**		
Less than good	**1.51**	(1.32–1.73)	*	**1.59**	(1.41–1.79)	*
**In your neighbourhood there are: sufficient green spaces**						
A lot or some	**1**			**1**		
None	**1.36**	(1.23–1.50)	*	**1.21**	(1.10–1.34)	*
**Noise from outdoors annoys you**						
None	**1**			**1**		
A lot or some	**0.98**	(0.89–1.09)	NS	**1.04**	(0.94–1.14)	NS
**Bad smells come into your house from outside**						
None	**1**			**1**		
A lot or some	**0.98**	(0.86–1.11)	NS	**1.04**	(0.93–1.17)	NS
**The air is highly polluted in your neighbourhood**						
None	**1**			**1**		
A lot or some	**0.87**	(0.76–1.00)	<0.05	**0.91**	(0.80–1.03)	NS
**Your neighbourhood is affected by an industry**						
None	**1**			**1**		
A lot or some	**0.86**	(0.72–1.03)	NS	**1.03**	(0.86–1.22)	NS
**Quality of your neighbourhood environment**						
Good	**1**			**1**		
Bad	**0.95**	(0.84–1.07)	NS	**1.04**	(0.93–1.17)	NS
**Size of municipality**						
<10,000	**1**			**1**		
10,000–100,000	**0.95**	(0.84–1.09)		**1.04**	(0.92–1.18)	
>100,000	**0.87**	(0.76–0.99)	0.01	**1.06**	(0.93–1.20)	NS
**Economic level of municipality**						
Low	**1**			**1**		
Medium	**0.84**	(0.73–0.95)	*	**0.98**	(0.86–1.11)	NS
High	**0.79**	(0.68–0.91)		**0.91**	(0.79–1.05)	
**Unemployment in municipality**						
Low	**1**			**1**		
Medium	**1.11**	(0.99–1.26)	NS	**1.16**	(1.03–1.30)	0.02
High	**1.08**	(0.96–1.22)		**1.17**	(1.04–1.32)	
**Illiteracy in municipality**						
Low	**1**			**1**		
Medium	**1.27**	(1.12–1.43)	*	**1.26**	(1.12–1.42)	*
High	**1.33**	(1.18–1.51)		**1.18**	(1.04–1.33)	

NS: Not significant (>0.05);

** p < 0.05;

*: P < 0.001.

**Table 3.** Multivariate Analysis. Odds ratio for sedentariness and variables related to characteristics of individuals and their physical.

		**Men**			**Women**	
**Model 1.Characteristics of Individuals**	**OR**	**95% CI**	**P**	**OR**	**95% CI**	**P**
**Educational Level**						
Graduate	**1**			**1**		
Secondary	**1.16**	(0.95–1.42)		**1.25**	(1.02–1.53)	
Primary	**1.83**	(1.51–2.21)		**1.50**	(1.23–1.84)	
No studies	**2.46**	(1.90–3.17)	*	**2.71**	(2.07–3.55)	*
**Occupational Class**						
Class I (highest)						
Class II	**1.04**	(0.86–1.25)	NS	**1.03**	(0.86–1.25)	0.01
Class III	**1.17**	(0.98–1.39)		**1.25**	(1.05–1.48)	
Class IV (lowest)	**1.17**	(0.91–1.50)		**1.32**	(1.03–1.68)	
**Employment status**						
Student	**1**			**1**		
Employed	**1.82**	(1.43–2.30)	*	**1.50**	(1.18–1.89)	0.01
Unemployed	**1.45**	(1.11–1.90)		**1.18**	(0.91–1.53)	
Retired / disabled	**1.11**	(0.80–1.53)		**1.27**	(0.87–1.84)	
Homemaker				**1.33**	(1.01–1.75)	
**Children ≤ 15 years**						
No	**1**			**1**		
Yes	**1.16**	(0.99–1.36)	NS	**1.10**	(0.94–1.29)	NS
**Marital status**						
Single	**1**			**1**		
Married / couple	**1.16**	(0.97–1.40)	NS	**0.91**	(0.74–1.11)	NS
Separated, divorced, widowed	**1.19**	(0.88–1.62)		**0.87**	(0.65–1.16)	
**Obesity**						
No (<30)	**1**			**1**		
Yes (≥30)	**1.54**	(1.31–1.80)	*	**1.19**	(1.00–1.41)	NS
**Smoking status**						
Non-smoker	**1**			**1**		
Smoker	**1.75**	(1.54–1.99)	*	**1.19**	(1.04–1.36)	0.03
Ex-smoker	**1.01**	(0.85–1.18)		**0.96**	(0.76–1.20)	
**Self-rated Health**						
Good	**1**			**1**		
Less than good	**1.54**	(1.32–1.79)	*	**1.36**	(1.18–1.57)	*
**Model 2. Characteristics of the Physical Environment**						
**In your neighbourhood there are: sufficient green spaces**						
A lot or some	**1**			**1**		
None	**1.37**	(1.24–1.52)	*	**1.19**	(1.08–1.32)	*
**Bad smells come into your house from outside**						
None	**1**			**1**		
A lot or some	**1.09**	(0.93–1.27)	NS	**1.09**	(0.95–1.26)	NS
**The air is highly polluted in your neighbourhood**						
None	**1**			**1**		
A lot or some	**0.86**	(0.72–1.03)	NS	**0.80**	(0.68–0.94)	0.01
**Your neighbourhood is affected by an industry**						
None	**1**			**1**		
A lot or some	**0.94**	(0.76–1.17)	NS	**1.11**	(0.90–1.36)	NS
**Noise from outdoors annoys you**						
None	**1**			**1**		
A lot or some	**1.02**	(0.90–1.14)	NS	**1.05**	(0.93–1.17)	NS
**Quality of your neighbourhood environment**						
Good	**1**			**1**		
Bad	**0.94**	(0.82–1.08)	NS	**1.04**	(0.91–1.18)	NS
**Model 3. Characteristics of the Social Environment**						
**Size of municipality**						
<10,000	**1**			**1**		
10,000–100,000	**0.93**	(0.81–1.08)	NS	**1.03**	(0.90–1.19)	NS
>100,000	**0.85**	(0.72–1.02)		**1.03**	(0.87–1.23)	
**Economic level of municipality**						
Low	**1**			**1**		
Medium	**1.22**	(1.00–1.49)	NS	**1.12**	(0.92–1.36)	NS
High	**1.11**	(0.97–1.27)		**1.10**	(0.96–1.26)	
**Unemployment in municipality**						
Low	**1**			**1**		
Medium	**1.29**	(1.12–1.49)	*	**1.18**	(1.02–1.36)	NS
High	**1.02**	(0.88–1.18)		**1.11**	(0.96–1.28)	
**Illiteracy in municipality**						
Low	**1**			**1**		
Medium	**1.35**	(1.19–1.54)	*	**1.29**	(1.13–1.46)	*
High	**1.39**	(1.21–1.59)		**1.22**	(1.07–1.40)	

NS: Not significant (>0.05);

** p < 0.05;

*: P < 0.001.

**Table 4.** Multivariate model. Factors influencing sedentariness in the adult population, age-adjusted.

	**Men**			**Women**		
	**OR**	**95% CI**	**P**	**OR**	**95% CI**	**P**
**Educational Level**						
University	**1**			**1**		
Secondary	**1.19**	(0.97–1.46)	*	**1.24**	(1.01–1.53)	*
Primary	**1.88**	(1.55–2.28)		**1.48**	(1.21–1.81)	
No studies	**2.47**	(1.91–3.21)		**2.68**	(2.04–3.52)	
**Occupational Class**						
Class I (highest)	**1**			**1**		
Class II	**1.05**	(0.86–1.26)	NS	**1.05**	(0.87–1.27)	0.01
Class III	**1.20**	(1.00–1.43)		**1.26**	(1.06–1.49)	
Class IV (lowest)	**1.19**	(0.92–1.53)		**1.39**	(1.09–1.78)	
**Employment status**						
Student	**1**			**1**		
Employed	**1.93**	(1.52–2.45)	*	**1.47**	(1.17–1.85)	0.02
Unemployed	**1.52**	(1.15–2.00)		**1.19**	(0.92–1.54)	
Retired / disabled	**1.10**	(0.79–1.53)		**1.31**	(0.90–1.91)	
Homemaker				**1.31**	(1.01–1.70)	
**Children <15 years**						
No	**1**			**1**		
Yes	**1.23**	(1.07–1.42)	*	**1.05**	(0.91–1.21)	NS
**Obesity**						
No (<30)	**1**			**1**		
Yes (≥30)	**1.52**	(1.30–1.79)	*	**1.18**	(0.99–1.40)	NS
**Smoking status**						
Non-smoker	**1**			**1**		
Smoker	**1.77**	(1.55–2.01)	*	**1.19**	(1.04–1.37)	0.02
Ex-Smoker	**1.02**	(0.87–1.21)		**0.94**	(0.75–1.19)	
**Self-rated Health**						
Good	**1**			**1**		
Less than good	**1.55**	(1.33–1.81)	*	**1.36**	(1.17–1.58)	*
**In your neighbourhood there are sufficient green spaces**						
A lot or some	**1**			**1**		
None	**1.26**	(1.13–1.41)	*	**1.26**	(1.13–1.41)	*
**Unemployment in municipality**						
Low	**1**			**1**		
Medium	**1.01**	(0.88–1.16)	NS	**0.99**	(0.86–1.14)	NS
High	**0.86**	(0.74–0.99)		**0.88**	(0.76–1.02)	
**Illiteracy in municipality**						
Low	**1**			**1**		
Medium	**1.42**	(1.23–1.64)	*	**1.25**	(1.08–1.45)	0.01
High	**1.45**	(1.25–1.69)		**1.13**	(0.97–1.31)	

NS: Not significant (>0.05);

** p < 0.05;

*: P < 0.001.
